# Special Education Major or Attitudes to Predict Teachers' Self-Efficacy for Teaching in Inclusive Education

**DOI:** 10.3389/fpsyg.2021.680909

**Published:** 2021-06-30

**Authors:** Ghaleb H. Alnahdi, Susanne Schwab

**Affiliations:** ^1^Department of Special Education, College of Education, Prince Sattam Bin Abdulaziz University, Alkharj, Saudi Arabia; ^2^Centre for Teacher Education, University of Vienna, Vienna, Austria; ^3^Research Focus Area Optentia, North-West University, Vanderbijlpark, South Africa

**Keywords:** self-efficacy, Saudi Arabia, special education teachers, major, attitudes, inclusive education

## Abstract

This study aimed to determine the predictors for Saudi Arabian teachers' self-efficacy to work in inclusive education. Five independent variables were tested in this study: attitudes toward inclusive education, participants' educational major, having relative with disability, working with students with disability and gender. Further, predictors of teachers' attitudes toward inclusive education were examined. The sample was 185 elementary-school teachers in Saudi Arabia. The Arabic version of the Teacher Efficacy for Inclusive Practices scale was used to measure self-efficacy. To assess attitudes toward inclusion an Arabic version of the Sentiments, Attitudes, and Concerns about Inclusive Education Revised subscale was used. Results showed teacher attitude toward inclusion are strongly linked with teachers' self-efficacy to work in inclusive classrooms. Further, participants with a relative with a disability showed more positive attitudes for inclusive education. Levels of self-efficacy were unaffected by gender, having a special education degree, or having a relative with a disability. In sum, this study highlighted the importance of teachers' attitudes toward inclusive education as a main predictor of teachers' self-efficacy.

## Introduction

The inclusion of students with disabilities in regular schools has evolved rapidly over recent decades in Saudi Arabia (Alnahdi et al., 2019) as well as throughout the world (see e.g., Schwab, 2020 for Europe). This results in an increasing number of schools that include students with disabilities. Preparing teachers to work with students with disabilities has also been considered in Saudi Arabia, as evident by the expansion of the number of special education departments in universities.

One of the challenges of this major change in the education system is determining how to prepare teachers for their new responsibilities and to provide the best possible support for students with special education needs. In this context, it is important to examine what variables influence the implementation of inclusive education. Based on previous research, teachers' attitudes toward inclusive education (e.g., Avramidis and Norwich, 2002; De Boer et al., 2011; Pit-ten Cate et al., 2018; Schwab, 2018) and their level of self-efficacy to teach in inclusive education (e.g., Malinen and Savolainen, 2016; Yada et al., 2019; Savolainen et al., 2020) are important factors for successful inclusive education (see the review of Mieghem et al., 2018).

Theoretically, the assumption that teachers' attitudes toward inclusive education are highly important for the implementation quality of inclusive education, can be grounded using the theory of planned behavior (Ajzen and Fishbein, 1977; Ajzen, 1991). Within this theory the assumption was be made that teachers' behavior in inclusive classes is shaped by teachers' attitudes as well and perceived behavioral control (and perceived subjective norms), Also empirical evidence was already given indicating that more positive attitudes toward inclusive education are linked with more inclusive behavior of teachers (e.g., Hellmich et al., 2019; Schwab et al., 2019). Further, teachers' self-efficacy toward inclusive education can be interpreted as a component of perceived behavioral control. High self-efficacy means that teachers are highly confident about their skills to work in inclusive education. According to Bandura, self-efficacy refers to people's beliefs in their “capabilities to organize and execute the courses of action required to produce given attainments” (Bandura, 1997; p. 3). Similarly as for attitudes, also for teachers self-efficacy beliefs some empirical evidence was found that it predicts teachers' actual behavior (e.g., Ghaith and Yaghi, 1997; Milner, 2002; Ryan et al., 2015; Miller et al., 2017). Regarding the relationship between teachers' attitudes toward inclusive education and teachers' self-efficacy beliefs, several studies indicated a positive relation (e.g., Varcoe and Boyle, 2014; Desombre et al., 2018; Saloviita, 2018; Yada et al., 2018; Miesera et al., 2019; Savolainen et al., 2020).

### Predictors of Teachers' Self-Efficacy and Attitudes Toward Inclusive Education

As possible predicators for teachers' attitudes toward inclusive education, teachers sociodemographic characteristics, such as gender and age, have been investigated frequently. However, for self-efficacy beliefs this was less frequently the case This might be related to the fact that, according to Tschannen-Moran and Hoy (2007), “demographic variables have typically not been strong predictors of the efficacy beliefs of teachers” (p. 952). In general, the existing literature provides no conclusions on whether gender effects teachers' self-efficacy. Some studies indicate that female teachers have a higher self-efficacy than male teachers (e.g., Tait and Mundia, 2013), while other studies (e.g., Tschannen-Moran and Hoy, 2007; Antoniou et al., 2017; Schwab et al., 2017) found no differences or even contrary results [higher self-efficacy for male teachers see e.g., Sak (2015)]. Interestingly, in terms of teachers' attitudes toward inclusive education, a clear gender effect has been shown; the review of De Boer et al. (2011) and a more recent overview by Schwab (2018) indicate that female teachers tend to hold a more positive attitude toward inclusive schooling than their male counterparts.

Next to teachers' gender, teachers' contact with students with special needs has been examined in previous studies. Although contact might be associated with teachers' attitudes toward inclusive schooling [for an overview see, e.g., Schwab (2018)], there is little evidence of the connection between teachers' contact with students with special needs and teachers' self-efficacy. For example, Schwab et al. (2017) found that contact has no effect on teachers' self-efficacy. However, Leyser et al. (2011) found that contact with children with special educational needs has a positive effect on teachers' self-efficacy.

In addition to teachers' gender, and previous contact experiences, also teachers professional experiences/familiarity with inclusive education and/or special needs education has been studied as predictors of both, teachers' self-efficacy as well as their attitudes. For instance, Gebhardt et al. (2015) demonstrated that special education teachers (in special schools) have higher self-efficacy than regular teachers (in mainstream settings). Similarly, Sharma et al. (2015) found that majoring in special education is a significant predictor of self-efficacy. Also, Leyser et al. (2011) and Schwab (2019) confirmed higher self-efficacy in special needs teachers than regular teachers.

In general (preservice), teachers' self-efficacy beliefs have been intensively studied worldwide: Australia (Sharma et al., 2012), China (Malinen et al., 2012), Europe [see, e.g., Schwab et al. (2017) for Austria and Germany; Savolainen et al. (2012) for Finland], Japan (Yada and Savolainen, 2017; Yada et al., 2018), Pakistan (Sharma et al., 2015), Saudi Arabia (Alnahdi, 2019, 2020a), and South Africa (Savolainen et al., 2012). Vieluf et al. (2013) found in a cross-national study of 23 countries that teachers' self-efficacy varies between countries because countries differ in cultural value orientation and teachers from different countries might vary in their response styles. Similarly, teachers' attitudes has been studies in several countries and for instance the cross-cultural study of Leyser et al. (1994) indicated country specific differences in the results. Educational policies in inclusive education and the implementation of these policies vary widely across countries (Schwab, 2020). Therefore, it seems to be important to investigate teachers' self-efficacy as well as their attitudes toward inclusive education in a culture specific way.

### Research Hypothesis

Although many studies have examined teachers' self-efficacy in different countries, there is little research from Saudi Arabia. As teachers' self-efficacy seems to be culturally influenced, this study aims to investigate if the predictors summarized in the introduction predict Saudi Arabian teachers' self-efficacy. Moreover, the present study will explicitly examine predictors of teachers' attitudes toward inclusive education and investigate whether the influence of predictors is similar for both constructs.

Based on the literature review, the following hypotheses are proposed:

- Higher self-efficacy beliefs of teachers to work in inclusive education are associated with more positive attitudes toward inclusive education.
- Female teachers have a more positive attitude toward inclusive education than male teachers.
- Teachers' attitudes toward inclusive education are positively influenced by previous contact with students with special needs.
- Teachers who majored in special education have higher perceived self-efficacy to work in inclusive education than other teachers.
- Teachers who majored in special education express more positive attitudes toward inclusive education than regular teachers.

In addition to the hypotheses above, the influence of gender and contact with students with special needs on teachers' self-efficacy will also examined. However, no concrete hypothesis was developed based on the previous literature.

## Materials and Methods

### Sample and Data

The study's participants were 214 teachers from eight elementary schools in the city of Riyadh, Saudi Arabia. After excluding questionnaires in which <50% of the items were answered (29), 185 participants were included in the analysis. Participation was voluntary, and the teachers had no obligation to complete the questionnaires. Approximately 15% of participants included in the analysis were female teachers and 85% were male. Teachers who majored in special education made up 40% of the participants, while the remaining 60% were teachers with other educational backgrounds. Around 47% of the sample have relative with disability, and around 44% are working with students with disabilities.

### Instruments

#### Teachers' Self-Efficacy to Work in Inclusive Education

The Teacher Efficacy for Inclusive Practices (TEIP) scale (see Appendix 1), developed by Sharma et al. (2012), was used to measure teachers' perceived self-efficacy to implement inclusive classroom practices. The TEIP scale (18 items) includes three domains that measure efficacy in managing behavior (EFMB subscale), efficacy in inclusive instruction (EFII subscale), and efficacy in collaboration (EFC subscale). Each domain contains six items. The scale's psychometrics were tested and approved for the Arabic version (Alnahdi, 2019, 2020a). For example, construct validity was tested by confirmatory factor analysis and acceptable fit indices were obtained (see Appendix 2). The goodness of fit index (GFI) was > 0.9, and the comparative fit index (CFI) was >0.9, indicating good fit (Hu and Bentler, 1998; Pugesek et al., 2003). In addition, the standardized root mean square (SRMR) was 0.0466, which indicates a good fit (Hu and Bentler, 1998, 1999; Schermelleh-Engel et al., 2003). A six-point Likert scale was used for each question item. A high score indicates a “high sense of perceived teaching efficacy for teaching in inclusive classrooms” (Sharma et al., 2012; p. 15).

#### Teachers' Attitudes Toward Inclusive Education

The attitude toward inclusions subscale from the Sentiments, Attitudes, and Concerns about Inclusive Education Revised Scale (SACIE-R) by Forlin et al. (2011) was used to assess teachers' attitudes. For the present study, however, one item was excluded from the original subscale (students who are inattentive should be in regular classes): the researchers believed that it might not be understood by participants as it was proposed in the English version because it could be interpreted in a way as to include many unintended students. A 4-point Likert scale was used for each item. A score close to the midpoint mean score (2.5) indicates that the teachers, on average, do not hold strong attitudes for or against inclusive education (Malinen et al., 2012).

The participants also completed a short demographic questionnaire to indicate their gender and college major. Teachers' contact with students with special needs/disabilities was obtained through two questions: do you have a relative with a disability (no/yes), and have you worked with students with disabilities (no/yes).

### Statistical Analysis

IBM SPSS Statistics 21 software was used for statistical analysis. First, Cronbach's alpha was calculated to determine the reliability of the TEIP scale as a whole, each of the three domains, and the attitudes subscale of the SACIE-R scale. Second, descriptive statistics—means and standard deviations—were calculated. Third, multiple regression analysis was performed to examine self-efficacy predictors. Fourth, analysis of variance (ANOVA) was conducted to examine any differences in self-efficacy and attitudes by teachers who majored in special education and other teachers. Fifth, multiple regression analysis was conducted to examine attitudes toward inclusive education predictors.

## Results

### Instruments and Descriptive Results

The internal consistency of the TEIP scale was examined by computing the Cronbach's alpha coefficient. The Cronbach's alpha reliability for the TEIP scale was 0.963. In addition, the Cronbach's alpha reliability for its three subscales (EFMB, EFII, and EFC) was 0.908, 0.907, and 0.896, respectively, which indicates very good consistency within each domain (George and Mallery, 2003). The Cronbach's alpha reliability for the attitudes subscale of SACIE was 0.725 (see Table 1).

**Table 1:** Means (M), standard deviations (SD), and reliability (Cronbach's alpha coefficients) of the TEIP and its three subscales, as well as the attitudes scale.

	***N***	**Alpha**	***M***	**SD**
TEIP (18 items)	185	0.963	4.55	1.15
Efficacy behavior (1-2-7-8-11-17)	185	0.908	4.51	1.22
Efficacy inclusive instruction (5-6-10-14-15-18)	185	0.907	4.64	1.19
Efficacy collaboration (3-4-9-12-13-16)	185	0.896	4.47	1.26
Attitudes toward inclusive education (four items)	147	0.725	2.53	0.71

According to teachers' self-efficacy, the mean scores all show a rather high value compared to the theoretical mean of the scale (3.5): all means of the three subscales, as well as the total mean score, are above 3.5. For teachers' attitudes, the mean score indicates a neutral attitude because the empirical mean is close to the theoretical mean of the scale (2.5).

### Predictors of Self-Efficacy

Table 2 shows that attitudes are the only significant predictor of teachers' self-efficacy. We performed multiple regression analysis (stepwise) with self-efficacy (TEIP) as the dependent variable and using all other variables as predictors (major, gender, relative with a disability, work experience with students with disabilities, and attitudes toward inclusion). The results showed that most of these independent variables do not significantly predict teachers' self-efficacy. The only significant model found that attitudes was a predictor, which explained around 28% of the variance (R^2^ = 0.28, *F*_(1, 112)_ = 9.61, *p* = 0.002).

**Table 2:** Multiple regression statistics to predict teachers' self-efficacy.

**Model**		**Unstandardized coefficients**		**Standardized coefficients**	***t***	**Sig**.
		***B***	**Std. error**	**Beta**		
1	(Constant)	3.427	0.378		9.078	0.000
	Attitudes	0.442	0.143	0.281	3.101	0.002

*Dependent Variable: TEIP_Overall*.

In the above-described regression analysis, college major had no effect on the overall TEIP score. However, to further examine whether teachers with a special education major showed higher self-efficacy than those with other majors on the three subscales, we used ANOVA. Results shown in Table 3 indicated that there are no statistically significant mean differences on the TEIP scale between teachers with a special education major and teachers with other majors. In addition, no statistically significant mean differences were detected for any of the three subscales. Moreover, no statistically significant mean differences were found on attitudes toward inclusion of students with disabilities based on major.

**Table 3:** Means/standard deviations and ANOVA statistics by teachers' major.

**SPED major**	**Yes**	**NO**	**Tests of between-subject effects**
	***N* (70)**	***N* (104)**	
	***M* (SD)**	***M* (SD)**	
Overall	4.68 (1.32)	4.50 (1.02)	*F*_(1, 172)_ = 0.976, *P* = 0.325
EFMB	4.64 (1.34)	4.48 (1.09)	*F*_(1, 172)_ = 0.787, *P* = 0.376
EFII	4.76 (1.37)	4.60 (1.06)	*F*_(1, 172)_ = 0.727, *P* = 0.395
EFC	4.60 (1.47)	4.43 (1.11)	*F*_(1, 172)_ = 0.737, *P* = 0.392
Attitudes	2.64 (0.80)	2.45 (0.63)	*F*_(1, 132)_ = 2.166, *P* = 0.143

*EFMB, efficacy in managing behavior; EFII, efficacy in inclusive instruction; EFC, efficacy in collaboration*.

### Predictors of Attitudes Toward Inclusive Education

We ran multiple regression analysis (stepwise) with attitudes as the dependent variable and by adding all other variables as predictors (major, gender, relative with a disability, self-efficacy, and work experience with students with disabilities). As shown in Table 4, the significant model with having a relative with a disability and self-efficacy as the predictors explained 17% of the variance in attitudes toward inclusive education (R^2^ = 0.17, *F*_(2, 111)_ = 11.46, *p* < 0.05). The other significant model with one predictor (having a relative with a disability) explained 12% of the variance in attitudes (R^2^ = 0.12, *F*_(1, 112)_ = 15, *p* < 0.05). Thus, we conclude that having a relative with a disability is the best single predictor in our set of variables. Second, adding self-efficacy to the model improved the explained variance by the model from 12% to around 17% (R^2^ increased to 0.17).

**Table 4:** Multiple regression coefficients to predict teachers' attitudes.

	**Model**	**Unstandardized coefficients**		**Standardized coefficients**	***t***	**Sig**.
		**B**	**Std. error**	**Beta**		
1	(Constant)	3.302	0.206		16.042	0.000
	Relative with disability	0.502	0.130	0.344	3.874	0.000
2	(Constant)	2.547	0.347		7.336	0.000
	Relative with disability	0.449	0.128	0.307	3.512	0.001
	TEIP_	0.148	0.056	0.233	2.665	0.009

*Dependent Variable: Attitudes*.

## Discussion

As teachers' self-efficacy beliefs are known as a crucial variable in implementing inclusive education, much research over the past decades has focused on this topic. However, this is the one of few studies to measure the self-efficacy and attitudes of teachers in Saudi Arabia using the TEIP and SACIE-R scales. As these scales have been used already in different countries, this study is a meaningful addition that allows direct comparisons between studies across cultures.

First, the results for the psychometric qualities of TEIP indicated high reliability, and the expected factorial structure was confirmed. As for the descriptive results, we found that Saudi Arabian teachers expressed high levels of self-efficacy in general: they were around 80% confident about their abilities to work in inclusive classrooms (with a mean around 4.5, in a 1-6 score range). Finnish teachers scored a similar TEIP mean (Savolainen et al., 2012). However, teachers in this study had a lower TEIP mean than teachers in Bangladesh (Ahsan et al., 2012) and in South Africa (Savolainen et al., 2012). Interestingly, there was little variance between the efficacy on the three subscales (efficacy in managing behavior, inclusive instruction, and collaboration). This finding is in contrast with some studies that found teachers scored lowest on self-efficacy in managing students' behavior (e.g., Yada and Savolainen, 2017). Generally, students' behavior problems are assumed to be challenging for teachers' self-efficacy (Spilt and Koomen, 2009). For instance, Schwab (2019) found that hyperactivity and inattention of students have an impact on teachers' self-efficacy.

Regarding teachers' attitudes toward inclusive education, the results of the present study show neutral to slightly positive attitudes toward inclusive education. For comparison, the Saudi Arabian teachers scored higher than Japanese teachers (Yada and Savolainen, 2017). However, this might be attributed to the culture-based response style, which increases approval rates with some items in some cultures more than others (Morren et al., 2011). Teachers' attitudes toward inclusive education was the only predictor of teachers' self-efficacy. This result is consistent with other studies' findings, confirming a positive relationship between positive attitudes toward inclusive education and high levels of self-efficacy (e.g., Sharma et al., 2012). Other variables examined in this study (having a relative with a disability, gender, major, or work experience with students with disability) did not significantly contribute to explaining teachers' self-efficacy. We expected that teachers who majored in special education in college would have greater self-efficacy to work with students with different abilities than teachers with other majors. Due to the fact that they are prepared specifically to work with students with disabilities, we assumed that those teachers would have a greater knowledge of teaching students with special needs. Moreover, they were expected to have more knowledge of legislation and policy, which can be a predictor of teaching efficacy for inclusive practices (Forlin et al., 2014). However, our findings did not show that teachers who majored in special education had a significantly greater self-efficacy than other teachers, either on the overall TEIP or on its three subscales.

This result is different from a previous findings from a study of Pakistani teachers (Sharma et al., 2015), which found that special education teachers expressed higher perceived self-efficacy. The lack of a difference in the self-efficacy between teachers who majored in special education and other teachers in Saudi Arabia might be explained by one of following reasons. Teachers in Saudi Arabia are currently influenced by the national movement to recognize the rights of all students to receive education in the schools located closest to their residences. This might close the gap in awareness that used to distinguish teachers who majored in special education from other teachers. Another explanation may relate to gender: the majority of teachers in this study were men, and a future study with more female teachers who majored in special education might produce more positive attitudes that would be consistent with different studies that found female from different range of ages, hold more positive attitudes toward peers with disabilities or toward inclusive education (Vignes et al., 2009; De Boer et al., 2012; Barakat, 2014; Schwab, 2018; Alnahdi et al., 2019; Alnahdi, 2020b) in comparison with their male counterparts.

## Conclusion and Implications

The results of this study underpin the importance of teachers' attitudes toward inclusive education as a main predictor of teachers' self-efficacy beliefs. Therefore, education officials should work on developing teachers' attitudes on two levels. First, this work should begin in special education departments in universities. Universities should consider applicants' attitudes toward people with disabilities in general and toward inclusive education more specifically when admitting students into special education programs. Second, raising teachers' awareness of disability rights of students can be used to influence teachers' self-efficacy to work in inclusive education, as has been found in prior studies.

### Limitations

To the best of our knowledge, this study is one of the first studies conducted in an Arabic country to explore the relationship between teachers' self-efficacy and attitudes toward inclusive education. However, there are a few limitations that must be considered in understanding the results. First, despite the effort to collect data from more teachers and cover many variables, given the time and other research constraints of this study, the study sample was not large enough to adequately detect differences based on our independent variables. Second, the scales used in this study were translated from English to Arabic. Although the translation process was carefully performed, it is possible that some of the items in the Arabic version did not preserve the essence of the original version. Therefore, future research should examine both versions to uncover any different meanings and ensure that the Saudi Arabian version of the TEIP is measurement invariant with respect to the original version. Third, the findings as to the relationships between the dependent variables and the examined predictors should be interpreted with caution in terms of generalization, and this study should be replicated with a different sample to ensure the generalisability of its findings.

## Data Availability Statement

The datasets generated for this study are available on request to the corresponding author.

## Ethics Statement

Ethical review and approval was not required for the study on human participants in accordance with the local legislation and institutional requirements. The patients/participants provided their written informed consent to participate in this study.

## Author Contributions

GA did collect and analyze the data and drafted the manuscript. SS participated in the introduction and the discussion sections. All authors contributed to the article and approved the submitted version.

## Conflict of Interest

The authors declare that the research was conducted in the absence of any commercial or financial relationships that could be construed as a potential conflict of interest.

## Appendix

**Appendix 1:** The teacher efficacy for inclusive practices (TEIP) scale (18 items).

**Domain**	**Item #**	
Efficacy in managing behavior	1	I can make my expectations clear about student behavior
	2	I am able to calm a student who is disruptive or noisy
	7	I am confident in my ability to prevent disruptive behavior in the classroom before it occurs
	8	I can control disruptive behavior in the classroom
	11	I am able to get children to follow classroom rules
	17	I am confident when dealing with students who are physically aggressive
Efficacy in inclusive instruction	5	I can accurately gauge student comprehension of what I have taught
	6	I can provide appropriate challenges for very capable students
	10	I am confident in designing learning tasks so that the individual needs of students with disabilities are accommodated
	14	I am confident in my ability to get students to work together in pairs or in small groups
	15	I can use a variety of assessment strategies (e.g., portfolio assessment, modified tests, performance-based assessment)
	18	I am able to provide an alternate explanation or example when students are confused
Efficacy in collaboration	3	I can make parents feel comfortable coming to school
	4	I can assist families in helping their children do well in school
	9	I am confident in my ability to get parents involved in the school activities of their children with disabilities
	12	I can collaborate with other professionals (e.g., itinerant teachers or speech pathologists) in designing educational plans for students with disabilities
	13	I am able to work jointly with other professionals and staff (e.g., aides, other teachers) to teach students with disabilities in the classroom
	16	I am confident in informing others who know little about laws and policies related to the inclusion of students with disabilities

**Appendix 2:** Goodness-of-fit indices for three-factor models of the TEIP scale.

**Model**	**SBS-χ^2^**	***p***	**df**	**RMSEA**	**CFI**	**SRMR**	**GFI**
M2	365.759	0.000	128	0.066	0.933	0.0424	0.910

*SBS-χ2, Satorra–Bentler scaled chi-squared; df, degrees of freedom; RMSEA, root–mean–square error of approximation; CFI, comparative fit index; SRMR, standardized root–mean–square residual; GFI, goodness-of-fit index*.

**Appendix 3:** Correlations.

	**TEIP**	**Attitudes**	**I'm currently working with SWD**	**Relative with disability**	**Gender**	**Major**
TEIP	1					
Attitudes	0.234^**^	1				
I'm currently working with SWD^a^	–0.043	–0.047	1			
Relative with disability	0.147	0.320^**^	-0.207^**^	1		
Gender	–0.038	0.064	–0.081	0.068	1	
Major	–0.090	–0.121	0.596^**^	–0.103	–0.008	1

** *Correlation is significant at the 0.01 level (2-tailed)*,

a *Students with disability*.

## Abbreviations

EFC: efficacy in collaboration
EFII: efficacy in inclusive instruction
EFMB: efficacy in managing behavior
SACIE-R: Sentiments, Attitudes and Concerns about Inclusive Education Revised Scale
TEIP: Teacher Efficacy for Inclusive Practices.
